# Study on the effect and mechanism of Zhenzhu Tongluo pills in treating diabetic peripheral neuropathy injury

**DOI:** 10.1186/s40001-024-01744-4

**Published:** 2024-03-01

**Authors:** Pengfei Dong, Lin Zhou, Xiaohui Wang, Lianping Xue, Yang Du, Rui Cui

**Affiliations:** 1 Department of Chinese Medicine, The Second Affiliated Hospital of Zhengzhou University, Zhengzhou, 450014 China; 2 Department of Pharmacy, The First Affiliated Hospital of Zhengzhou University, Zhengzhou, 450052 China; 3 Department of Ultrasound, The First Affiliated Hospital of Zhengzhou University, Zhengzhou, 450052 China; 4 Second Ward of Internal Medicine, Rehabilitation Hospital of Zhengzhou Cigarette Factory, Zhengzhou, 450000 China; 5 Department of Ultrasonography, The Sixth Affiliated Hospital, Sun Yat-Sen University, Guangzhou, 510000 China

**Keywords:** Zhenzhu Tongluo pills, Diabetic peripheral neuropathy, Neuroprotection, Hyperalgesia, Structural and functional damage

## Abstract

**Background:**

As a traditional Mongolian medicine, Zhenzhu Tongluo pills has played a good neuroprotective function in clinic. However, the key mechanisms by which it works are poorly studied.

**Objectives:**

To study the effect and mechanism of Zhenzhu Tongluo pills in treating diabetic peripheral neuropathy injury.

**Methods:**

Diabetic peripheral neuropathy model was established by injecting STZ into rats. Physiological, behavioral, morphological and functional analyses were used to evaluate that the overall therapeutic effect of rats, ELISA, qRT-PCR, Western blot, immunohistochemical staining, HE staining and TUNEL staining were used to further study the related mechanism.

**Results:**

Zhenzhu Tongluo pills can significantly improve the physiological changes, behavioral abnormalities, structural and functional damage in diabetic peripheral neuropathy rats, which may be related to the anti-inflammatory and anti-apoptotic effects that realized by regulating PI3K/AKT, MAPK, NF-κB signaling pathways.

**Conclusions:**

Zhenzhu Tongluo pills has neuroprotective effect, and anti-inflammatory and anti-apoptosis may be the important way of its function.

## Introduction

Peripheral neuropathy is a complication with a high incidence of diabetes, often involving the nervous system, and has no obvious symptoms at the beginning of the disease. With the aggravation of neuropathy, abnormal sensory function, limb numbness, and local tingling gradually appear, and further development may lead to foot ulcers, and amputation is required in severe cases, with a certain disability rate. It can cause great damage to patients’ physical and mental health and quality of life. Timely and effective treatment can slow down the progression of diabetic peripheral neuropathy. At present, although drug treatment of diabetic peripheral neuropathy can be seen in clinic, including hypoglycemic and trophoneurotic drugs, the efficacy is still limited.

As an important part of Chinese medicine in China, Mongolian medicine plays an irreplaceable role in the treatment of diseases, which is a traditional medicine that has gradually formed and developed in long-term medical practice. Zhenzhu Tongluo pills is a Mongolian medicine composed of a variety of drugs, including *Hyriopsis cumingii* (Pearl), *Gypsum Fibrosum* (Gypsum), *Carthamus tinctorius L.*(Safflower), *Fructus Gardeniae* (gardenia*)*, *Syzygium aromaticum L.* (Flos Caryophyllata*)*, *Myristica fragrans Houtt.* (Semen Myristicae)*, Amomum tsaoko Crevost et Lemarie* (amomum tsao-ko), *Myristica fragrans Houtt*. (Nutmeg), *Syzygium aromaticum L.* (Flos Caryophyllata), *Aquilariae Lignum Resinatum* (Rosewood Heart Wood), *Amomun kravanh Pierre ex Gagnep* (cardamom), *Cinnamomum cassia Presl* (Cassia Bark), the plant name has been checked with http://www.worldfloraonline.org) (2023.07.01). It has the effects of clearing heat, opening the orifice, and drying dampness. Gypsum clears heat and relieves fire, and stops bleeding; Safflower promotes blood circulation, and removes blood stasis; Flos Caryophyllata and Nutmeg dispel cold and relieve pain, and calm the mind; White cardamom promotes digestion, removes cold and dampness; amomum tsao-ko promotes digestion, relieves cold and dampness. Clinical treatment over the years has found that the use of Zhenzhu Tongluo pills alone or in combination with other treatments can significantly improve the symptoms of patients with cerebral infarction, contusion-induced bone diseases, ischemic stroke and chronic rotator cuff injuries [1–6], indicating its excellent neuroprotective function. Network pharmacological analysis of Zhenzhu Tongluo pills based on above-mentioned ingredients has found that PI3K–AKT, MAPK, NFκB and other signals may be important pathways for the function of Zhenzhu Tongluo pills. Therefore, it is necessary for us to further study the relevant mechanism of Zhenzhu Tongluo pills functions through disease animal models and molecular biological approaches.

In this study, animal model was established by STZ-induced diabetic peripheral neuropathy. Then, different doses of Zhenzhu Tongluo pills were used to treat diabetic peripheral neuropathy, and morphological and functional analysis, ELISA, qRT-PCR, Western blot, immunohistochemical staining, HE staining and other techniques were used to explore the key possible mechanisms of drug treatment. This study will provide a scientific reference for Mongolian medicine treatment of peripheral neuropathy.

## Materials and methods

### Materials

#### Experimental animals

Male adult SD rats weighing 200–230 g were purchased from Zhengzhou University Laboratory Animal Center. The rats were kept in the best laboratory conditions, with a 12-h light and dark cycle, and they had free access to water and food. Prior to all behavioral experiments, the experimental animals were acclimated for more than 30 min in the behavioral testing room. All behavioral experiments were completed between 9:00 am and 4:00 pm. The experimental protocol was approved by Ethics Committee of the Second Affiliated Hospital of Zhengzhou University (2022003).

#### Experimental drugs

**Fig. 1:**
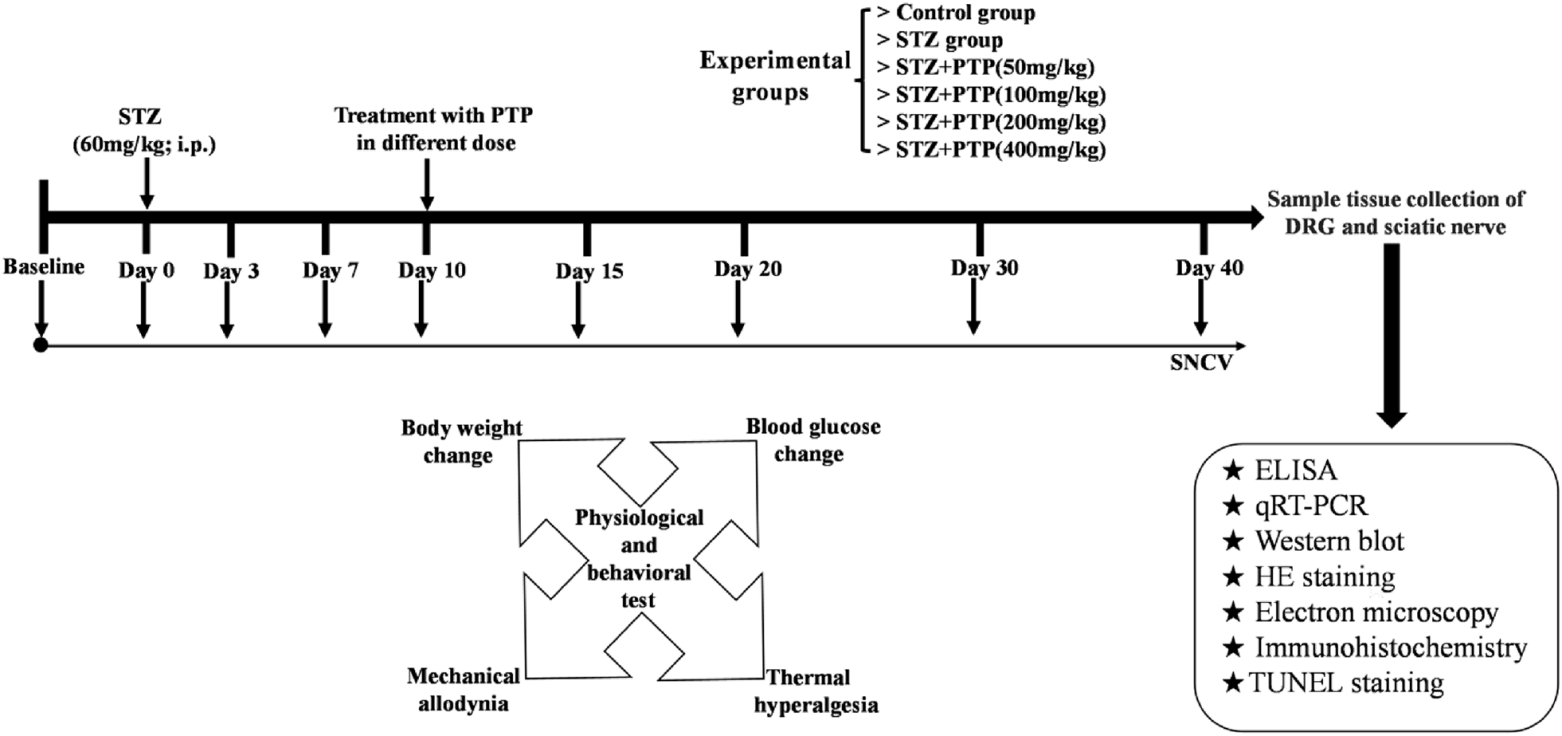
Research implementation plan diagram

(1) Zhenzhu Tongluo pills (INNER MONGOLIA OTAQI MONGOLIA MEDICINE COMPANY, INC.LTD) were administered by intragastric administration (Control group and STZ group were given normal saline intragastric administration); (2) Streptozotocin (STZ; MCE; HY-13753) was dissolved in citric acid buffer (pH = 4.4; 0.1 M), a single intraperitoneal injection of STZ (60 mg/kg) was used to induce diabetic peripheral neuropathy in rats. Timepoints of different drug administration were determined according to the timeline set in advance (Fig. 1).

#### Instrument

Ultraviolet spectrophotometer (752-P Shanghai Xianke Instrument Co., LTD), Electronic balance (PL-203 METTLER Toledo Instrument (Shanghai) Co., LTD), Table centrifuge (TGL-16c Shanghai Anting scientific Instrument factory), Cryocentrifuge (neofuge 15R heal force), Scanner (V300 EPSON), Gray analysis software (Image-Pro Plus), Image analysis software (Adobe PhotoShop, Adobe), Exposure meter (Tanon-4800 tanon).

#### Experimental reagent

RIPA lysate (P0013B Beyotime Biotechnology), PMSF (100 mM) (BL507A biosharp), Phosphorylase inhibitor (P1081 Beyotime Biotechnology), BCA protein quantitative detection kit (P0012 Beyotime Biotechnology), 5 × protein loading buffer (P0015 Beyotime Biotechnology), SDS–PAGE Gel preparation Kit (BL508A biosharp), Protein Marker (20350ES72 YEASEN), TRIS(1115GR500BIOFROXX), Glycine (1275KG2P5 BIOFROXX).

### Methods

#### Physiological indicators monitoring

0, 3, 7, 10, 15, 20 and 30 days after injection of STZ or citric acid buffer, the changes in body weight and fasting blood glucose (blood glucose measured with a glucometer by tail vein blood sampling) of each group of rats were observed.

#### Mechanical pain measurement

After adapting to the testing cage for about 30 min, each group of rats had their mechanical withdrawal threshold measured using Von Frey filaments (1–300 g). The hind paw of the rat was stimulated vertically by the filament and observed for withdrawal reflex after bending to an *S* shape. Five stimulations were performed and three positive responses were recorded as the mechanical withdrawal threshold. This behavioral test was conducted under double-blind conditions.

#### Thermal pain measurement

After adapting to the testing cage for 30 min, paw withdrawal latency of each group was measured using a thermal radiation instrument. The instrument parameters were set to achieve a withdrawal threshold of 10–15 s for normal rats to prevent burning of the paw. Each rat was subjected to 3 heat stimulations, and the average value was taken as the paw withdrawal latency. This behavioral test was conducted under double-blind conditions.

#### Sensory nerve conduction velocity (SNCV)

Before the rats were euthanized, their sensory nerve conduction velocity was measured. The experimental rats were anesthetized (with ketamine 75 mg·kg^−1^ and xylazine 7.5 mg·kg^−1^ intraperitoneal injection, both drugs inhibited the central nervous system and had no effect on peripheral nerve conduction), and their limbs were fixed on the experimental table in a prone position. The room temperature was maintained at 30 ℃. The sciatic nerve was bluntly separated and moistened with paraffin oil. A recording electrode was placed near the proximal end of the sciatic nerve, and a stimulating electrode was placed near the distal end for electric stimulation (Square wave, 1.5 V). The time (T) required for the stimulus to be transmitted from the distal electrode to the proximal electrode was recorded, and the distance (S) between the electrodes was measured to calculate the SNCV = S/T.

#### qRT-PCR detection

Total RNA from the dorsal root ganglion (DRG; L4–L6) was extracted using RNAiso and its concentration and purity were determined by spectrophotometry. The total RNA was quantified according to the instructions in the kit and then reverse transcribed into cDNA. Real-time quantitative PCR was performed using SYBR Green Master Mix. The primer sequences were as follows: (1) GAPDH forward, 5′-GTT CTA GAG ACA GCC GCA TCTTC-3′ and reverse, 5′-CAC TTT GTC ACA AGA GAA GGC AG-3′; (2) TNF-α forward, 5′-CTC AAG CCC TGG TAT GAG CC-3′ and reverse, 5′-GGC TGG GTA GAG AAC GGA TG-3′; (3) IL-1β forward, 5′-AAA TGC CTC GTG CTG TCT GA-3′ and reverse, 5′-AGG CCA CAG GGA TTT TGT CG-3′; (4) IL-6 forward, 5′-GTT GCC TTC TTG GGA CTG AT and reverse, 5′-TGT GTA ATT AAG CCT CCG ACT. Amplification was performed using a program of 95 °C (2 min) → [95 °C (15 s) → 60 °C (15 s) → 72 °C (1 min)] (44 cycles). The relative expression levels of the target gene were calculated based on GAPDH levels.

#### ELISA detection of TNF-α, IL-1β, and IL-6 expression levels in rat sciatic nerve

Following the experiment, rats were anesthetized by 2% isoflurane combined with 10% chloral hydrate intraperitoneally. Fresh DRG samples (L4–L6) were collected, added to RIPA lysis buffer, and tissue was homogenized by sonication or magnetic bead under icy conditions. The supernatant was collected after centrifugation at 12000 rpm for 10 min at 4 °C. ELISA kits (Elabscience; TNF-α: E-EL-R2856c; IL-1β: E-EL-R0012c; IL-6: E-EL-R0015c) were used to detect the concentrations of TNF-α, IL-1β, and IL-6 in the DRG tissue using an indirect immunoassay.

#### Western blotting

After rats were narcotized by 2% isoflurane combined with 10% chloral hydrate intraperitoneally, the DRG (L4–L6) of rats was extracted according to the method of inverse nerve walking, and quickly frozen at − 80 ℃ for later use. Lysate containing protease inhibitor was added to the tissue. The tissue was broken by ultrasound, centrifuged at 12000 rpm for 15 min at 4 ℃, and the supernatant was obtained. According to the instructions of the BCA kit, the protein concentration of each tissue was quantified, and 5 × Loading Buffer was added to adjust the protein concentration to the same level. After denaturing by metal bath at 100 ℃ for 5 min, the protein was isolated by 7% SDS–PAGE electrophoresis, and then was transferred to PVDF membrane. After being sealed with 5% BSA solution at room temperature for 1 h, PVDF membrane was transferred to primary antibody at 4℃ overnight (GAPDH: Ms, 1: 2000, Servicebio, GB15002; PI3K: Rb, 1: 1000, Proteintech, 20,584-1-AP; p-PI3K: Rb, 1: 1000, Affinity, AF3242; AKT: Ms, 1: 20,000, Proteintech, 60,203-2-Ig; p-AKT: Ms, 1: 20,000, Proteintech, 66,444-1-Ig; ERK: Rb, 1:10,000, Proteintech, 11,257-1-AP; p-ERK: Rb, 1:1000, Servicebio, GB11004; P38: Rb, 1:1000, Proteintech, 14,064-1-AP; p-P38: Rb, 1:1000, Servicebio, GB113380; P65: Rb, 1:1000, Proteintech, 10,745-1-AP; p-P65: Rb, 1:10,000, Proteintech, 82,335-1-RR; JNK: Rb, 1:5000, Proteintech, 17,572-1-AP; p-JNK: Rb, 1:2000, Proteintech, 80,024-1-RR; c-FOS: Rb, 1:1000, Servicebio, GB114125). After cleaning with TBST, PVDF membrane was transferred to secondary antibody (HRP Goat Anti Ms; 1: 5000, Servicebio, GB23301; HRP Goat Anti Rb; 1: 5000, Servicebio, GB23303) and incubated at room temperature for 2 h. After TBST cleaning, the bands were exposed in the chemical gel imager, and the gray value was analyzed by ImageJ.

#### The expression level of p-AKT in situ detected by immunohistochemistry

The paraffin-embedded tissues (DRG; L4–L6) were prepared into sections  5 μm thick, and immunohistochemical detection was performed using Roche automatic multifunctional pathological tissue detection system: (1) Bake the slices at 75 ℃ for 4 min; Dewaxing paraffin sections with special EZ-prep; Repair solution CC1(alkaline) was added, and the section heating plate with independent temperature control was heated to 100 ℃ for high temperature repair for 30 min. Add IU INHIBITOR 37 ℃ and incubate for 4 min. The sample feeder added 100 μl primary antibody (1:200 dilution), and the slices were heated to the set temperature of 95 ℃, and incubated for 16 min. The sample feeder was added with 100 μl HRP UNIVMULT and incubated at 37 ℃ for 8 min. 100 μl UVDAB and 100 μl UVDABH202 were added to the sample feeder and incubated at 37 ℃ for 8 min. Add 100 μl UV COPPER into the sample feeder and incubate at 37 ℃ for 4 min. HEMATOXY–LINII was added to 100 μl in the sample filler for re-staining and incubated for 4 min. (1) 100 μl BLUING REAGENT was added into the sampler and incubated for 4 min; (2) remove slices and wash slices with water containing mild detergent; (3) gradient ethanol dehydration; and (4) use xylene to clear the slice and seal the slice. PBS solution was used as negative control instead of primary antibody. Known positive sections were used as positive controls.

#### HE staining

After fixation with 4% PFA for 48 h, sciatic nerve were dehydrated and transparent with gradient alcohol and xylene, and preserved by paraffin embedding. Paraffin sections with a thickness of 5 μm were roasted in an oven at 70 ℃ for 2 h and dewaxed and hydrated with xylene and gradient alcohol. HE staining kit was used to stain the nucleus and cytoplasm of the sample tissue, and then, the sample was sealed with neutral resin, observed under microscope, and retained by photograph.

#### TUNEL staining

The DRG tissue (L4–L6) was embedded in paraffin with a thickness of 5 microns. After dewaxing hydration, repairing with protease K and breaking with 0.1% triton, the TUNEL staining kit (Servicebio; G1501) was used for coloring, and the cell nuclei were re-dyed with DAPI. The samples were sealed with anti-fluorescence quenching sealing reagent, and the images were observed and collected under a fluorescence microscope.

#### Electron microscopy of sciatic nerve

Sciatic nerve samples less than 1 mm^3^ were fixed by immersion in 0.1 M phosphate buffer (PH7.4) with 2.5% glutaraldehyde at 4 ℃ for 6 h, washed three times with 0.1 M PBS for 15 min each time, and then fixed in 1% osmium tetroxide for 2 h. The samples were dehydrated in gradient ethanol for 10 min each time, embedded in epoxy propane for 30 min, immersed in pure embedding solution at 37 ℃ for 2–3 h, and solidified in the embedding agent at 4 ℃ for 2–3 days. Ultra-thin slices of 50–70 nm thickness were obtained and stained with uranyl acetate and lead citrate for 30 min each, and observed and photographed under a LEO 906e transmission electron microscope.

### Statistical analysis

The results were statistically analyzed by using GraphPad Prism 8.3.0. All data are presented as the mean ± SEM. With regard to blood glucose, body weight, mechanical allodynia and thermal hyperalgesia, two-way analysis of variance (ANOVA) with repeated measures followed by Tukey’s post hoc test was used. One-way ANOVA followed by Tukey’s post hoc test was conducted for other data as appropriate. Differences were considered significant when a *P* value less than 0.05.

## Results

### STZ (60 mg/kg) can successfully induce diabetic peripheral neuropathy in rat

To evaluate the effect of Zhenzhu Tongluo pills on diabetic peripheral neuropathy, a rat model of diabetic peripheral neuropathy was induced by a single intrapitoneal injection of STZ (60 mg/kg), and verified by detecting fasting blood glucose, body weight, mechanical pain and heat pain.

#### Persistent reduced body weight gain and increased blood glucose are the significant physiological features of STZ rats

**Fig. 2:**
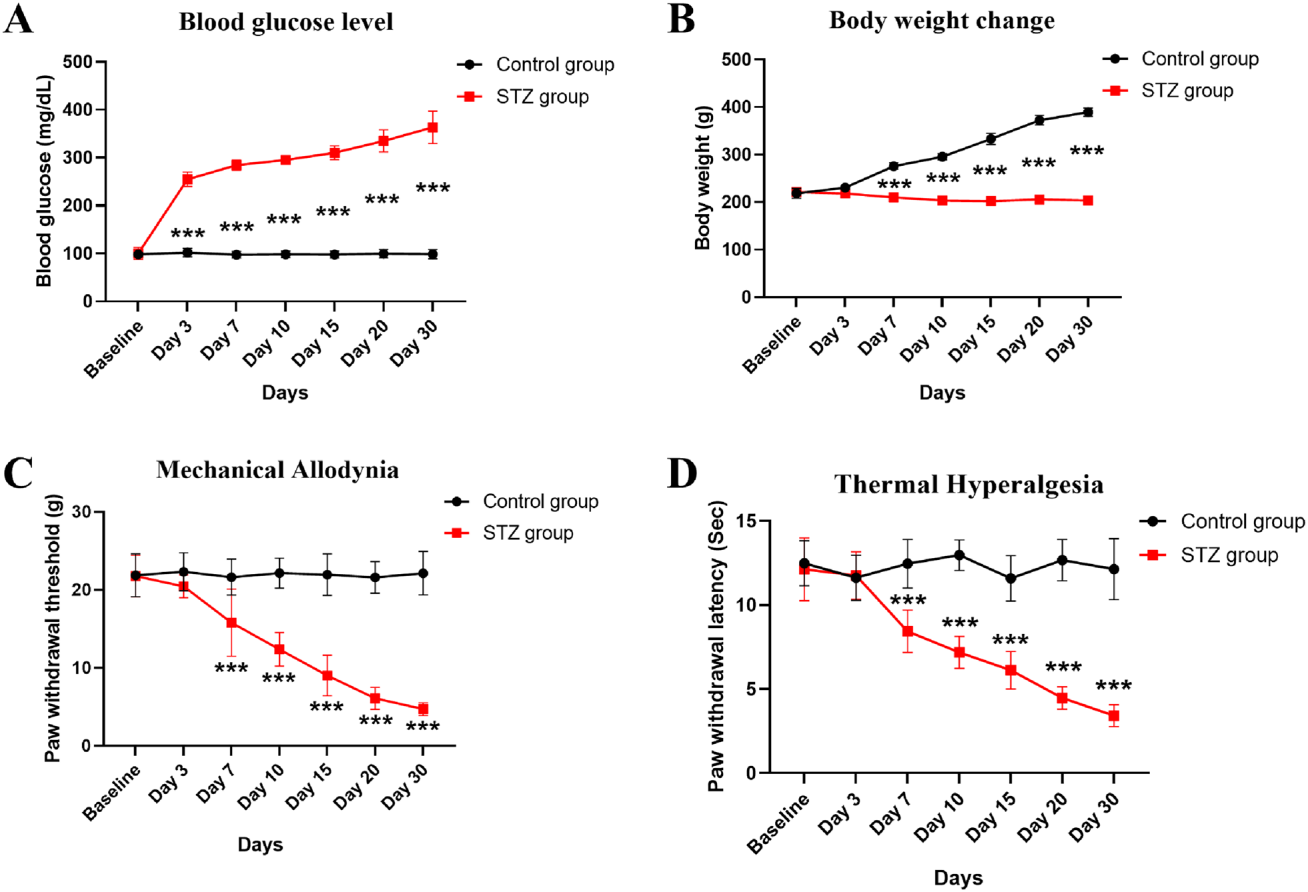
Single intraperitoneal injection of STZ can successfully induce diabetic peripheral neuropathy in a rat model. **A** Blood glucose level of STZ group was significantly increased compared with Control group (*n =* 6; ^***^p < 0.001 vs Control group); **B** body weight of STZ group was significantly decreased compared with Control group (*n =* 6; ^***^p < 0.001 vs Control group); **C** mechanical withdrawal threshold of rats in STZ group was significantly lower than that in Control group (*n =* 6; ^***^p < 0.001 vs Control group); **D** paw withdrawal latency of rats in STZ group was significantly decreased compared with Control group (*n =* 6; ^***^p < 0.001 vs Control group)

Compared with the Control group, fasting glucose levels in the STZ group were significantly increased at day 3, day 7, day 10, day 15, day 20 and day 30 after injection (Fig. 2A,* n* = 6, two-way ANOVA, ^***^p < 0.001 vs Control group). In contrast, the STZ group had significantly lower body weight at day 7, day 10, day 15, day 20, and day 30 after injection compared to the Control group (Fig. 2B, * n* = 6, two-way ANOVA, ^***^p < 0.001 vs Control group). Since high blood glucose levels and loss of weight gain are prominent features of diabetes, the above results indicate that the rat model of hyperglycemia has been successfully constructed from physiological indicators.

#### Mechanical and thermal hyperalgesia of STZ rats suggest the success of STZ-induced diabetic peripheral neuropathy in rats

In addition to conventional physiological indicators, mechanical withdrawal threshold and thermal withdrawal latency were evaluated before STZ injection and at 3 days, 7 days, 10 days, 15 days, 20 days and 30 days after STZ injection. We found that the mechanical withdrawal threshold and thermal withdrawal latency were significantly lower in the STZ group at day 7, day 10, day 15, day 20, and day 30 after injection than that in the Control group (Fig. 2C, D, * n* = 6, two-way ANOVA, ^***^p < 0.001 vs Control group), showing significant hyperalgesia. These results confirm the successful construction of diabetic peripheral neuropathy rat model.

### Zhenzhu Tongluo pills can effectively improve the reduced body weight gain and hyperalgesia of diabetic peripheral neuropathy rats

#### Zhenzhu Tongluo pills can effectively improve the reduced body weight gain of diabetic peripheral neuropathy rats, but has no significant effect on the increased blood glucose

**Fig. 3:**
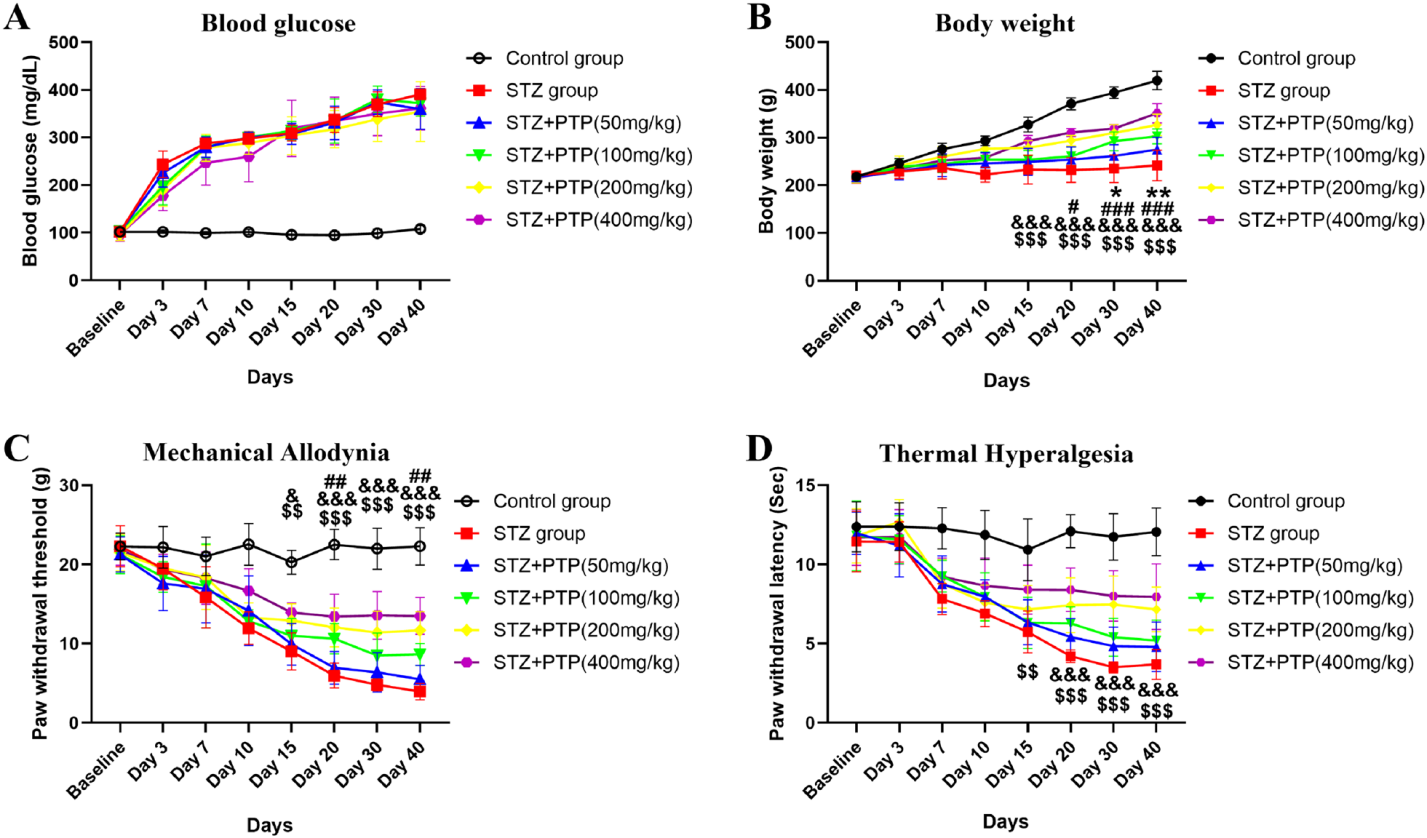
Zhenzhu Tongluo pills can significantly improve the physiological indexes and sensory dysfunction in diabetic peripheral neuropathy rats. **A** Zhenzhu Tongluo pills has no significant improvement effect on blood glucose level in diabetic peripheral neuropathy rats; **B** Zhenzhu Tongluo pills can improve the decreased body weight gain in diabetic peripheral neuropathy rats (*n =* 8, ^*^p < 0.05, ^**^p < 0.01: STZ + PTP (50 mg/kg) vs STZ group; ^#^*p* < 0.05, ^###^*p* < 0.001: STZ + PTP (100 mg/kg) vs STZ group; ^&&&^*p* < 0.001: STZ + PTP (200 mg/kg) vs STZ group; ^$$$^*p* < 0.001: STZ + PTP (400 mg/kg) vs STZ group); **C** Zhenzhu Tongluo pills significantly improved the mechanical allodynia of diabetic peripheral neuropathy rats (*n =* 8, ^##^*p* < 0.01: STZ + PTP (100 mg/kg) vs STZ group; ^&^*p* < 0.05, ^&&^*p* < 0.001: STZ + PTP (200 mg/kg) vs STZ group; ^$$^*p* < 0.01, ^$$$^*p* < 0.001: STZ + PTP (400 mg/kg) vs STZ group); **D** thermal hyperalgesia of diabetic peripheral neuropathy rats was significantly relieved by Zhenzhu Tongluo pills (*n =* 8; ^&&&^*p* < 0.001: STZ + PTP (200 mg/kg) vs STZ group; ^$$^*p* < 0.01, ^$$$^*p* < 0.001: STZ + PTP (400 mg/kg) vs STZ group)

While exploring the neuroprotective effect of Zhenzhu Tongluo pill on diabetic peripheral neuropathy rats, our study also focused on its influence on physiological indexes of diabetic peripheral neuropathy rats. Compared with STZ group, different doses of Zhenzhu Tongluo pills had no significant effect on the increased blood glucose induced by STZ (Fig. 3A). On the other hand, different doses of Zhenzhu Tongluo pills can significantly improve the decreased body weight gain of diabetic peripheral neuropathy rats at different time points. Compared with STZ group, STZ + PTP (50 mg/kg) group showed increased body weight gain at Day 30 and 40 after STZ injection (Fig. 3B, * n* = 8, two-way ANOVA, ^*^p < 0.05, ^**^p < 0.01 vs STZ group). STZ + PTP (100 mg/kg) group showed significant body weight gain from Day 20 after STZ injection (Fig. 3B, * n* = 8, two-way ANOVA, ^#^*p* < 0.05, ^###^*p* < 0.001 vs STZ group). STZ + PTP (200 mg/kg) and STZ + PTP (400 mg/kg) groups showed significant body weight gain from Day 15 after STZ injection (Fig. 3B, * n* = 8, two-way ANOVA, ^&&&^*p* < 0.001: STZ + PTP (200 mg/kg) vs STZ group; ^$$$^*p* < 0.001: STZ + PTP (400 mg/kg) vs STZ group. These results suggest that Zhenzhu Tongluo pills can improve part physiological indexes of STZ-induced diabetic peripheral neuropathy rats.

#### Zhenzhu Tongluo pills can effectively alleviate mechanical and thermal hyperalgesia in STZ-induced diabetic peripheral neuropathy rats

Neuroprotective function of Zhenzhu Tongluo pills was studied by evaluating its effect on pain sensitization in STZ-induced diabetic peripheral neuropathy rats. For mechanical pain, we found that different doses of Zhenzhu Tongluo pills significantly improved mechanical pain sensitization in STZ-induced rats. Compared with STZ group, STZ + PTP (100 mg/kg) group significantly improved mechanical pain sensitization after Day 20 of STZ injection (Fig. 3C, * n* = 8, two-way ANOVA, ^##^*p* < 0.01 vs STZ group). STZ + PTP (200 mg/kg) group and STZ + PTP (400 mg/kg) group showed relief on mechanical pain sensitization after Day 15 of STZ injection (Fig. 3C, n = 8, two-way ANOVA, ^&^*p* < 0.001, ^&&&^*p* < 0.001: STZ + PTP (200 mg/kg) vs STZ group; ^$$^*p* < 0.01, ^$$$^*p* < 0.001: STZ + PTP (400 mg/kg) vs STZ group). Similarly, we found that high doses of Zhenzhu Tongluo pills (200 mg/kg and 400 mg/kg) significantly improved thermal hyperalgesia in STZ-induced rats (Fig. 3D, * n* = 8, two-way ANOVA, ^&&&^*p* < 0.001: STZ + PTP (200 mg/kg) vs STZ group; ^$$^*p* < 0.01, ^$$$^*p* < 0.001: STZ + PTP (400 mg/kg) vs STZ group). The above results indicated that Zhenzhu Tongluo pills had a significant improvement on hyperalgesia in diabetic peripheral neuropathy rats, which suggested that Zhenzhu Tongluo pills has a better neuroprotective function in behavioral performance.

### Zhenzhu Tongluo pills can effectively improve the sensory conduction of sciatic nerve in diabetic peripheral neuropathy rats

**Fig. 4:**
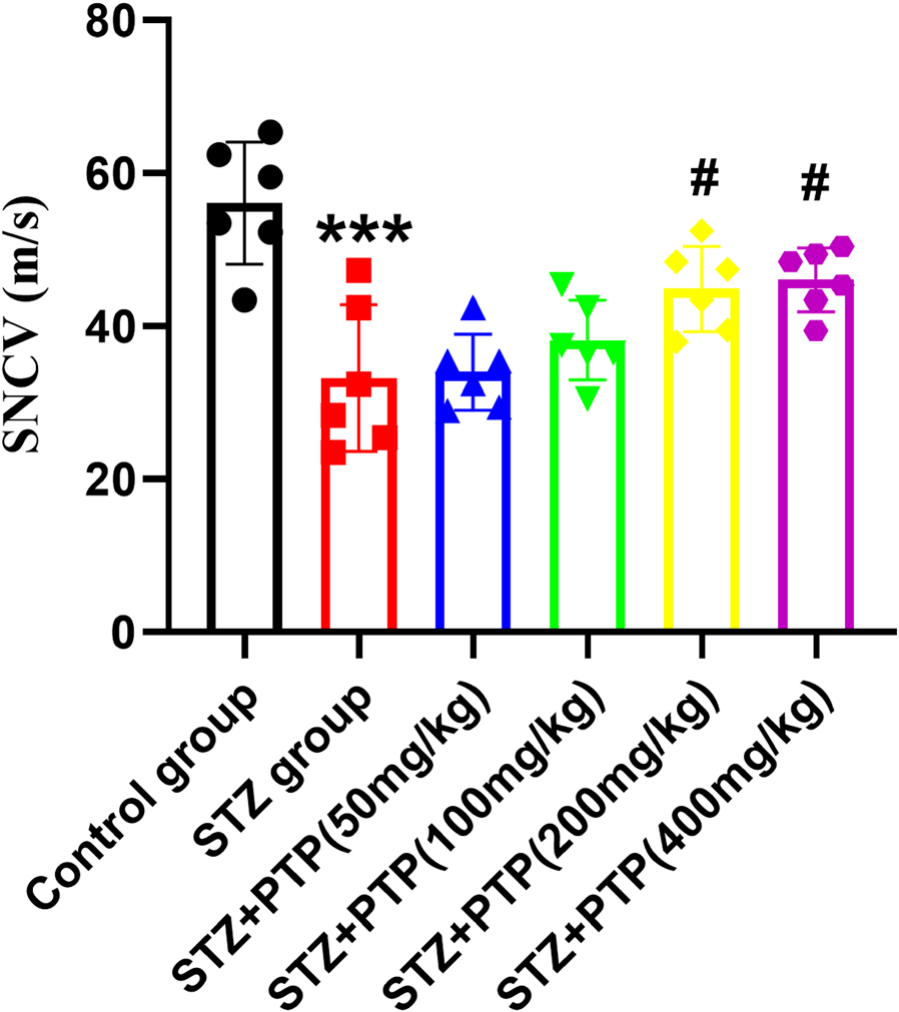
Zhenzhu Tongluo pills can significantly improve the abnormal sensory nerve conduction function in diabetic peripheral neuropathy rats. (*n =* 6, ^***^p < 0.001 vs Control group; ^#^*p* < 0.05 vs STZ group)

In fact, abnormal sensory conduction function of sciatic nerve is one of the important features of diabetic peripheral neuropathy rats. By examining the conduction velocity of sensory nerve under different treatment factors, we found that the conduction velocity of sensory nerve in the STZ group was significantly reduced compared with the Control group (Fig. 4, *n =* 6, one-way ANOVA, ^***^p < 0.001 vs Control group). After treatment with Zhenzhu Tongluo pills, we found that high-dose Zhenzhu Tongluo pills (200 mg/kg and 400 mg/kg) significantly improved the reduced sensory nerve conduction velocity in diabetic rats (Fig. 4, *n =* 6, one-way ANOVA, ^#^*p* < 0.001 vs STZ group). The above results confirm the neuroprotective effect of Zhenzhu Tongluo pills functionally.

### Zhenzhu Tongluo pills can improve the high expression of inflammatory factors in DRG of diabetic peripheral neuropathy rats

#### Zhenzhu Tongluo pills reduce the mRNA expression of TNF-α, IL-1β and IL-6 in DRG of diabetic peripheral neuropathy rats

**Fig. 5:**
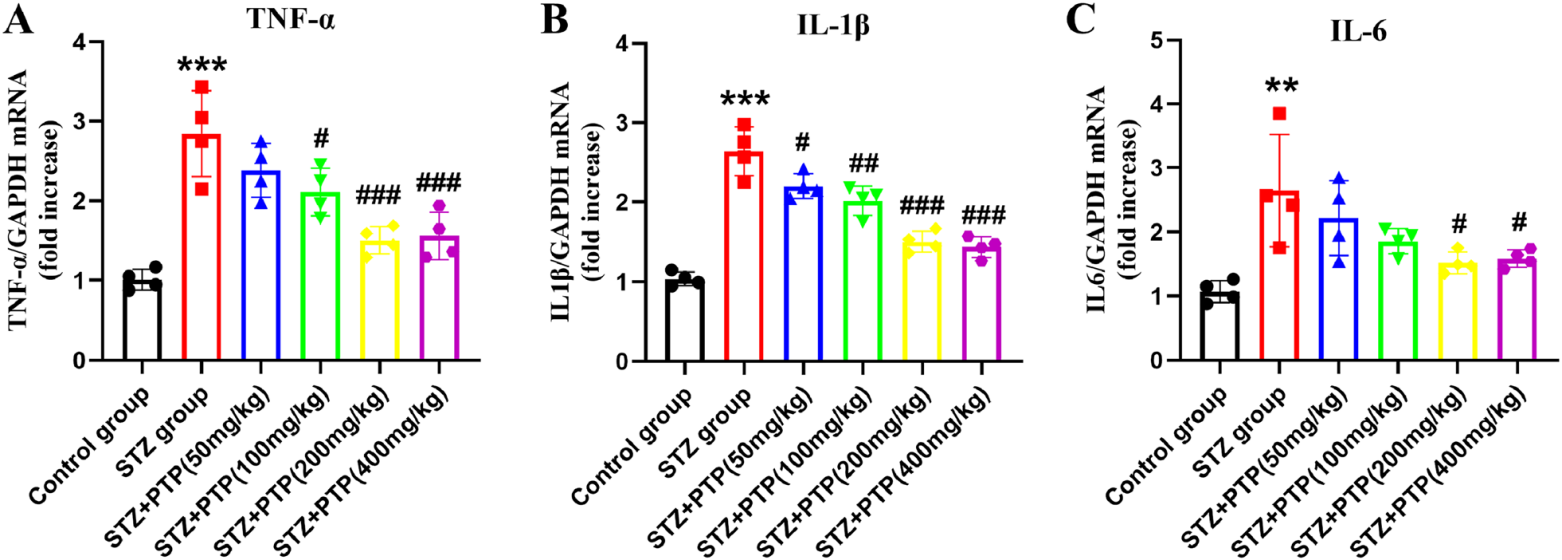
Zhenzhu Tongluo pills can significantly improve the increased expression of inflammatory factors at the mRNA level in DRG of diabetic peripheral neuropathy rats. **A** Zhenzhu Tongluo pills can significantly improve the expression of TNF-α on mRNA level in DRG of diabetic peripheral neuropathy rats (*n =* 4, ^***^p < 0.001 vs Control group; ^#^*p* < 0.05, ^###^*p* < 0.001 vs STZ group); **B** Zhenzhu Tongluo pills could significantly improve the expression of IL-1β on mRNA level in DRG of diabetic peripheral neuropathy rats (*n =* 4, ^***^p < 0.001 vs Control group; ^#^*p* < 0.05, ^##^*p* < 0.01, ^###^*p* < 0.001 vs STZ group); **C** Zhenzhu Tongluo pills could significantly improve the expression of IL-6 on mRNA level in DRG of diabetic peripheral neuropathy rats (*n =* 4, ^**^p < 0.01 vs Control group; ^#^*p* < 0.05 vs STZ group)

Excessive inflammatory response is associated with the occurrence and development of many diseases. Compared with normal rats, the high expression of pro-inflammatory factors in various tissues of diabetic peripheral neuropathy rats is one of the key factors of pain sensitization. In this study, by constructing an STZ-induced diabetic peripheral neuropathy rat model, we found that the mRNA expression of TNF-α, IL-1β and IL-6 in DRG of STZ rats was significantly increased compared with that in the Control group (Fig. 5, *n =* 4, one-way ANOVA, ^**^p < 0.01, ^***^p < 0.001 vs Control group). This confirmed the increased expression of inflammatory factors in DRG of diabetic peripheral neuropathy rats at mRNA level. By treating diabetic peripheral neuropathy rats with different doses of Zhenzhu Tongluo pills, we found that different doses of Zhenzhu Tongluo pills significantly improved the high expression of inflammatory factors at mRNA level in DRG of STZ rats (Fig. 5, *n* = 4, one-way ANOVA, ^#^*p* < 0.05, ^##^*p* < 0.01, ^###^*p* < 0.001 vs STZ group).

#### Zhenzhu Tongluo pills reduce the protein expression of TNF-α, IL-1β and IL-6 in DRG of diabetic peripheral neuropathy rats

**Fig. 6:**
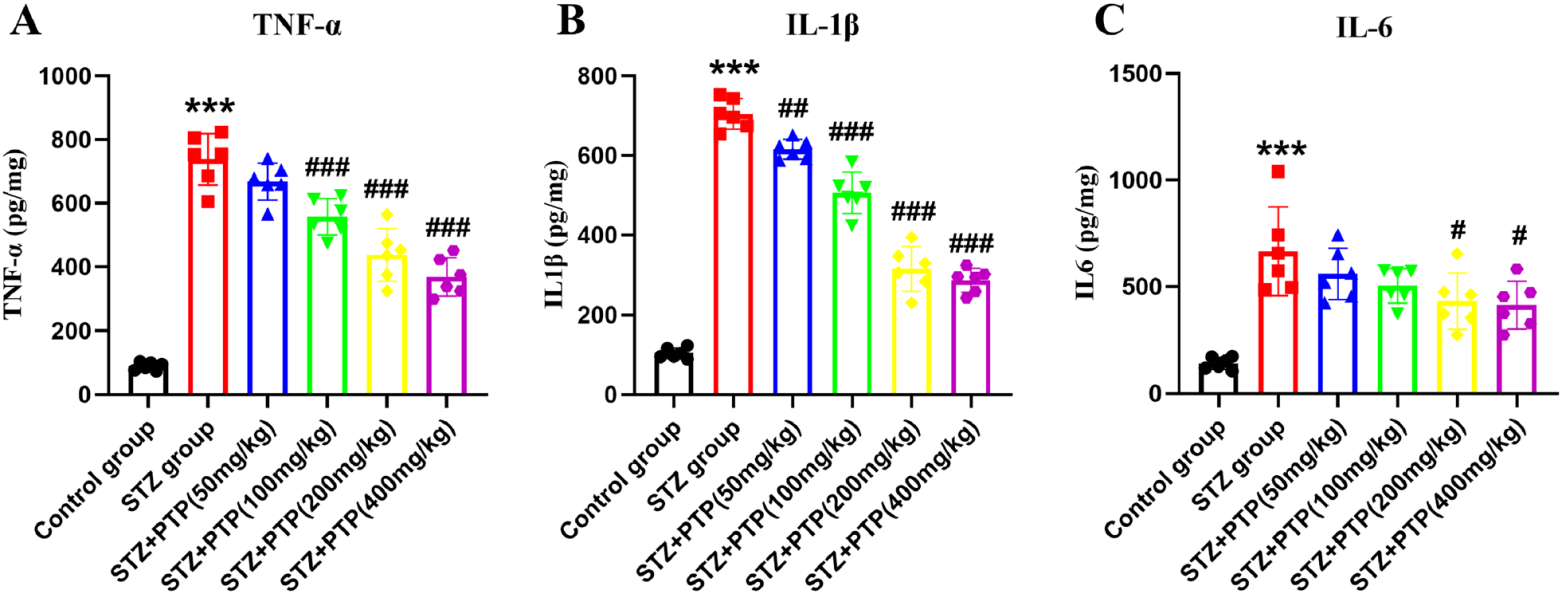
Zhenzhu Tongluo pills can significantly improve the increased expression of inflammatory factors at the protein level in DRG of diabetic peripheral neuropathy rats. **A** Zhenzhu Tongluo pills can significantly improve the expression of TNF-α in the protein level of DRG in diabetic peripheral neuropathy rats (*n =* 6, ^***^p < 0.001 vs Control group; ^###^*p* < 0.001 vs STZ group); **B** Zhenzhu Tongluo pills can significantly improve the expression of IL-1β in DRG of diabetic peripheral neuropathy rats (*n =* 6, ^***^p < 0.001 vs Control group; ^##^*p* < 0.01, ^###^*p* < 0.001 vs STZ group); **C** Zhenzhu Tongluo pills can significantly improve the expression of IL-6 in DRG of diabetic peripheral neuropathy rats (*n =* 6, ^***^p < 0.001 vs Control group; ^#^*p* < 0.05 vs STZ group)

After determining the expression of inflammatory factors at the mRNA level, we then detected the expression of TNF-α, IL-1β and IL-6 at the protein level by ELISA. Compared with Control group, the expression of inflammatory cytokines TNF-α, IL-1β and IL-6 in DRG in STZ group was significantly increased at the protein level (Fig. 6, *n* = 6, one-way ANOVA, ^***^p < 0.001 vs Control group). After treatment with different doses of Zhenzhu Tongluo pills, we found that the expression of TNF-α, IL-1β and IL-6 in DRG in the treatment group was significantly reduced compared with that in the STZ group (Fig. 6, *n* = 6, one-way ANOVA, ^#^*p* < 0.05, ^##^*p* < 0.01). ^###^*p* < 0.001 vs STZ group).

The above results confirmed that Zhenzhu Tongluo pills has a significant anti-inflammatory effect at the mRNA level and protein level, and it can effectively improve the increased inflammatory response in the DRG of STZ-induced diabetic peripheral neuropathy rats.

### Zhenzhu Tongluo pills modulate the expression of several key signaling molecules in DRG of diabetic peripheral neuropathy rats

#### Zhenzhu Tongluo pills can regulate the expression of PI3K–AKT pathway protein in DRG of diabetic peripheral neuropathy rats

**Fig. 7:**
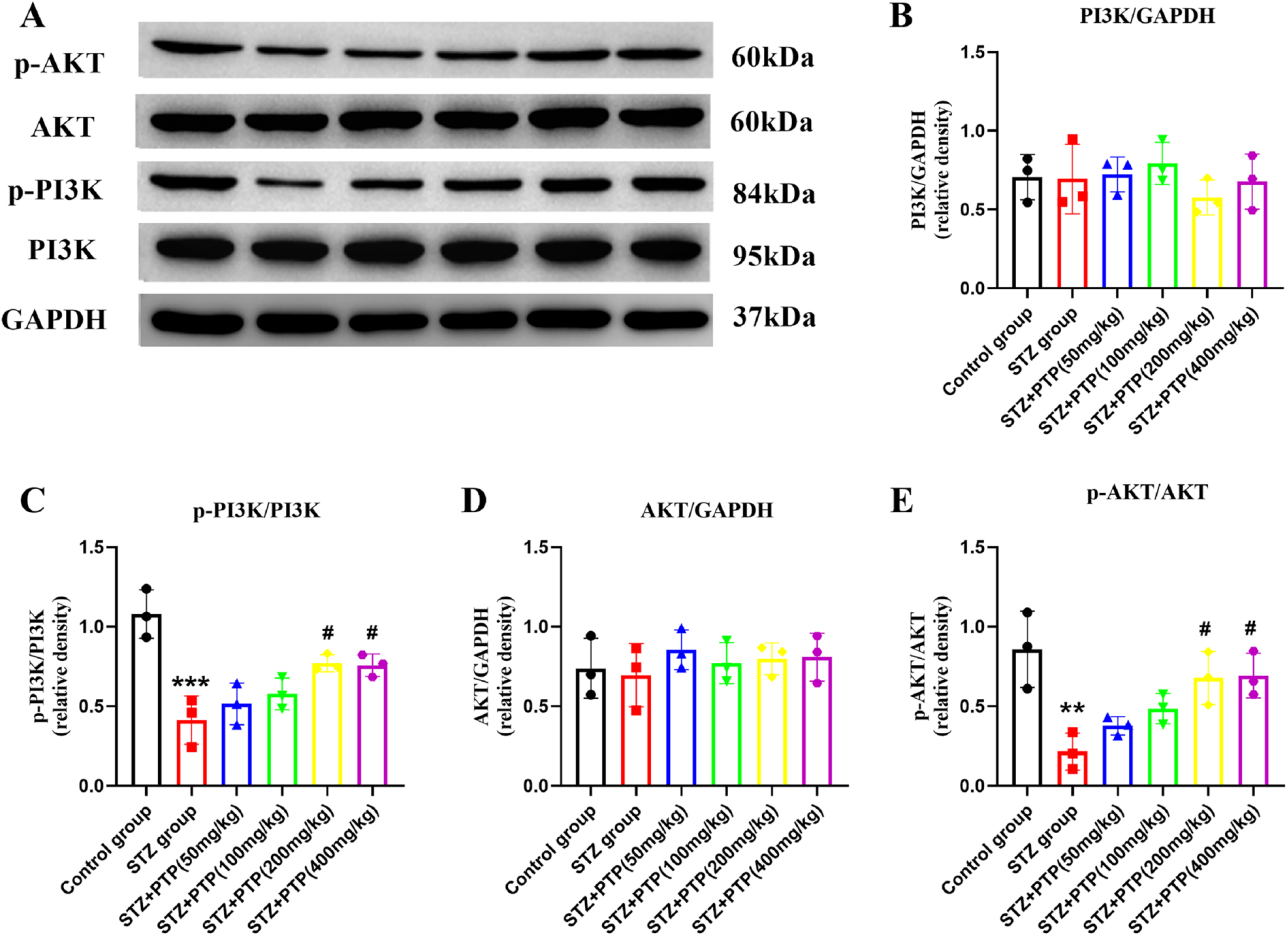
Effect of Zhenzhu Tongluo pills on the expression of protein molecules in PI3K–AKT signaling pathway in DRG of diabetic peripheral neuropathy rats. **A**, **B** There was no significant change in the expression of total PI3K protein in DRG among all groups (*n =* 3); **A**, **C** Zhenzhu Tongluo pills could significantly improve p-PI3K expression in DRG of diabetic peripheral neuropathy rats (*n =* 3, ^***^p < 0.001 vs Control group; ^#^*p* < 0.05 vs STZ group); **A**, **D** There was no significant change in the expression of total AKT in DRG among all groups (*n =* 3). **A**, **E** Zhenzhu Tongluo pills could significantly improve the reduced expression of p-Akt in DRG of diabetic peripheral neuropathy rats (*n =* 3, ^**^p < 0.01 vs Control group; ^#^*p* < 0.05 vs STZ group)

The expression changes of PI3K, p-PI3K, AKT and p-AKT in DRG of rats in different groups were detected by WB. Our results showed that no significant changes of total PI3K and AKT were found among different groups (Fig. 7A, B, D), but the expression of p-PI3K and p-AKT were significantly lower (Fig. 7A, C, E,* n* = 3, one-way ANOVA, ^**^p < 0.01, ^***^p < 0.001 vs Control group) than Control group; And the expression of p-PI3K and p-Akt were increased to varying degrees after treatment with different doses of Zhenzhu Tongluo pills (Fig. 7A, C, E,* n* = 3, one-way ANOVA, ^#^*p* < 0.05 vs STZ group). These results suggest that Zhenzhu Tongluo pills can regulate the expression of PI3K–AKT pathway protein molecules.

#### Immunohistochemical staining showed that Zhenzhu Tongluo pills could significantly improve the low expression of p-AKT in situ in DRG of STZ rats

**Fig. 8:**
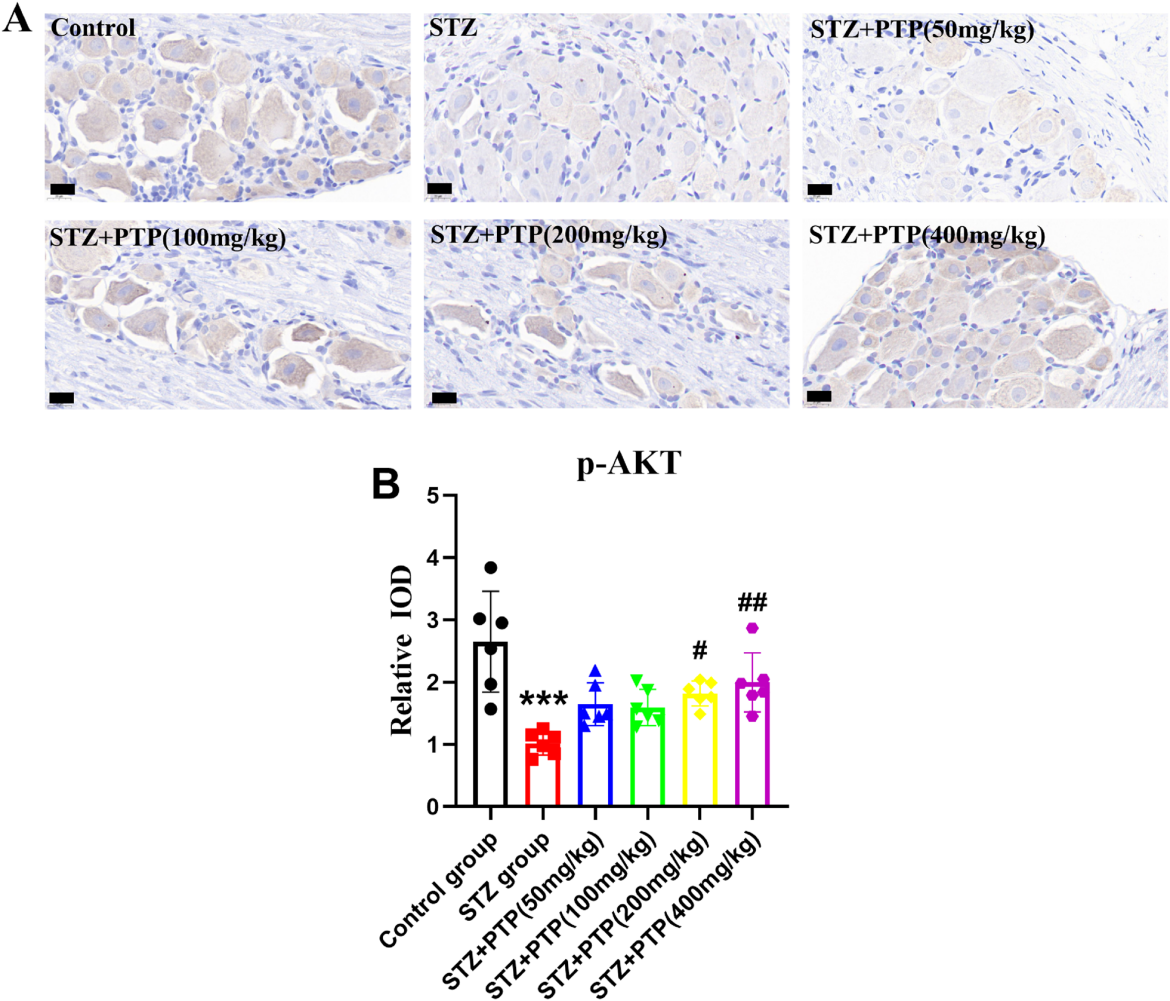
Immunohistochemical staining showed that Zhenzhu Tongluo pills could improve the decreased expression of p-Akt in DRG of diabetic peripheral neuropathy rats (*n =* 3, ^***^p < 0.001 vs Control group; ^#^*p* < 0.05, ^##^*p* < 0.01 vs STZ group; Scale bar = 20 μm)

The expression of p-AKT in different groups of DRG was detected in situ by immunohistochemistry. Compared with the Control group, the expression of p-Akt in STZ group was significantly reduced (Fig. 8A, B,* n* = 6, one-way ANOVA, ^***^p < 0.001 vs Control group). After treatment with different doses of Zhenzhu Tongluo pills, the expression of p-Akt was increased to varying degrees (Fig. 8A, B,* n* = 6, one-way ANOVA, ^#^*p* < 0.05, ^##^*p* < 0.01 vs STZ group).

#### Zhenzhu Tongluo pills can regulate the expression of NF-κB p65 and p38 MAPK protein in DRG of diabetic peripheral neuropathy rats

**Fig. 9:**
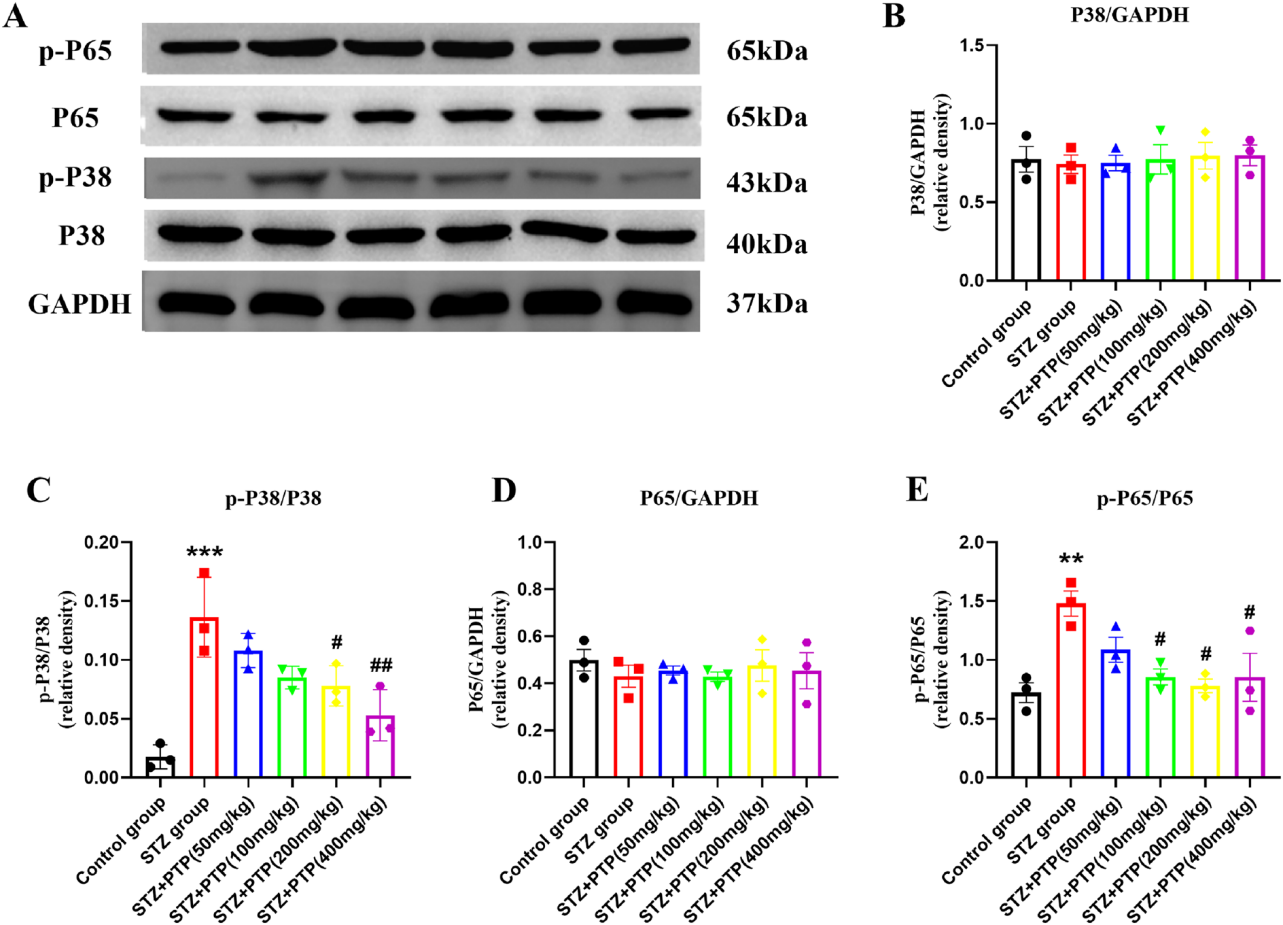
Effect of Zhenzhu Tongluo pills on the expression of NF-κB p65 and p38 MAPK protein in DRG of diabetic peripheral neuropathy rats. **A**, **B** There was no significant change in the expression of p38 MAPK protein in DRG among all groups (*n =* 3). **A**, **C** Zhenzhu Tongluo pills can significantly improve the increased expression of p-P38 MAPK in DRG of diabetic peripheral neuropathy rats (*n =* 3, ^***^p < 0.001 vs Control group; ^#^*p* < 0.05, ^##^*p* < 0.01 vs STZ group); **A**, **D** There was no significant change in the expression of total NF-κB p65 protein in DRG among all groups (*n =* 3). **A**, **E** Zhenzhu Tongluo pills can significantly improve the increased expression of p-NF-κB p65 in DRG of diabetic peripheral neuropathy rats (*n =* 3, ^**^p < 0.01 vs Control group; ^#^*p* < 0.05 vs STZ group)

In addition to the PI3K–AKT signaling pathway, we also detected the expression of molecular proteins related to the MAPK and NF-κB signaling pathways. Compared with the Control group, total protein expression of NF-κB p65 and p38 MAPK in each group had no significant change (Fig. 9A, B, D, *n* = 3), but p–p38 MAPK and p-NF-κB p65 expression were significantly increased (Fig. 9A, C, E,* n* = 3, one-way ANOVA, ^**^p < 0.01, ^***^p < 0.001 vs Control group); The expression of p–p38 MAPK and p-NF-κB p65 decreased to varying degrees after treatment with different doses of Zhenzhu Tongluo pills (Fig. 9A, C, E, *n* = 3, one-way ANOVA, ^#^*p* < 0.05, ^##^*p* < 0.01 vs STZ group).

#### Zhenzhu Tongluo pills can regulate the expression of ERK and JNK in DRG of diabetic peripheral neuropathy rats

**Fig. 10:**
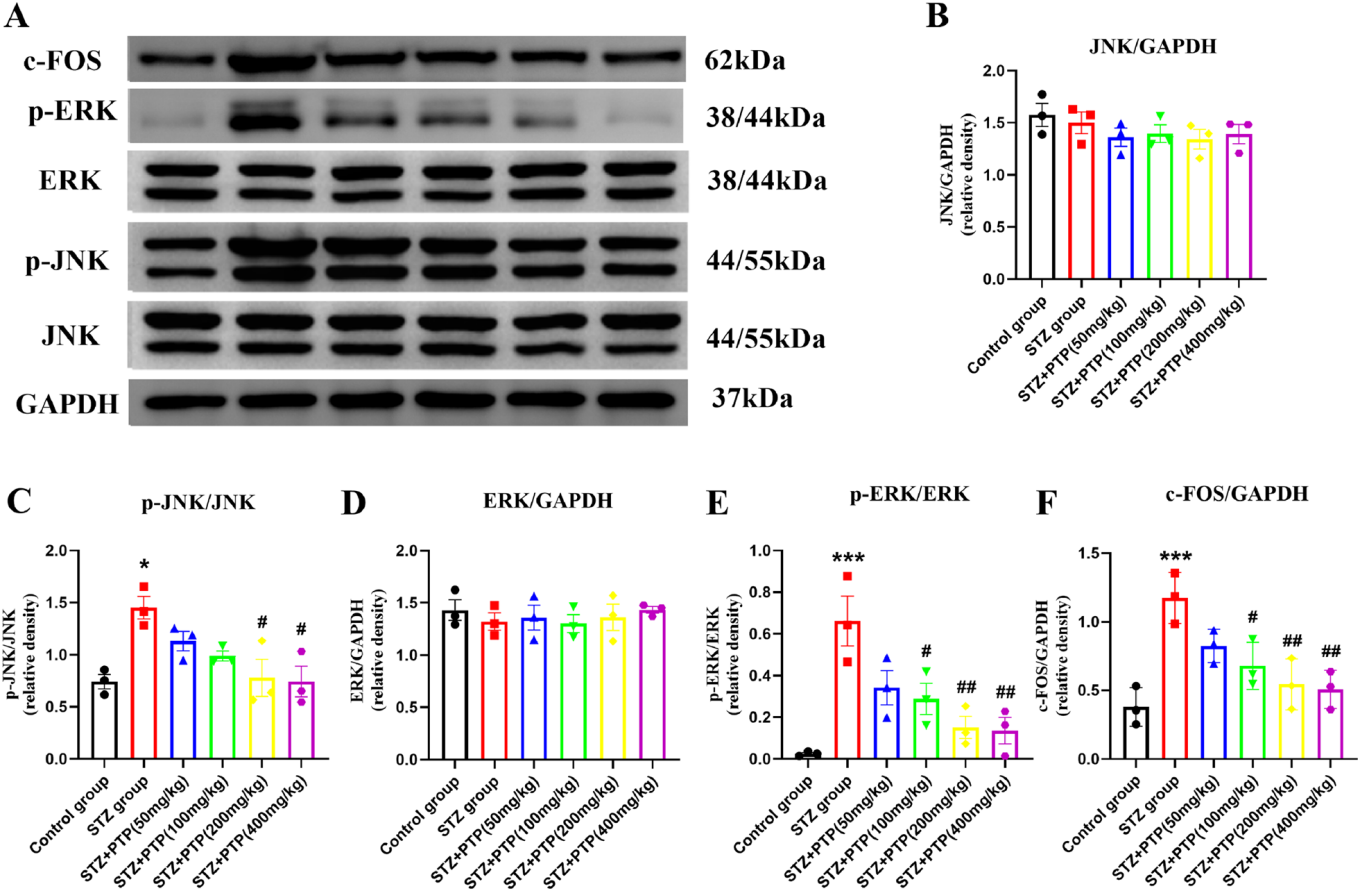
Effect of Zhenzhu Tongluo pills on the expression of JNK and ERK proteins in DRG of diabetic peripheral neuropathy rats. **A**, **B** There was no significant change in the expression of p38 MAPK protein in DRG among all groups (*n =* 3). **A**, **C** Zhenzhu Tongluo pills could significantly improve the increased expression of p-JNK in DRG of diabetic peripheral neuropathy rats (*n =* 3, ^*^p < 0.05 vs Control group; ^#^*p* < 0.05 vs STZ group); **A**, **D** There was no significant change in the expression of total ERK protein in DRG among all groups (*n =* 3). **A**, **E** Zhenzhu Tongluo pills could significantly improve the increased expression of p-ERK in DRG of diabetic peripheral neuropathy rats (*n =* 3, ^***^p < 0.001 vs Control group; ^#^*p* < 0.05, ^##^*p* < 0.01 vs STZ group); A,** F**: Zhenzhu Tongluo pills could significantly improve the increased expression of c-FOS in DRG of diabetic peripheral neuropathy rats (*n =* 3, ^***^p < 0.001 vs Control group; ^#^*p* < 0.05, ^##^*p* < 0.01 vs STZ group)

Compared with the Control group, the total protein of JNK and ERK in all groups were not significantly changed (Fig. 10A, B, D,* n* = 3), while the expression of p-ERK and p-JNK in STZ group were significantly increased (Fig. 10A, C, E,* n* = 3, one-way ANOVA, ^*^p < 0.05, ^***^p < 0.001 vs Control group). The expression of p-ERK and p-JNK were decreased to varying degrees after treatment with different doses of Zhenzhu Tongluo pills (Fig. 10A, C, E,* n *= 3, one-way ANOVA, ^#^*p* < 0.05, ^##^*p* < 0.01 vs STZ group). In addition, different doses of Zhenzhu Tongluo pills can also improve the expression of c-Fos in DRG of diabetic rats to varying degrees (Fig. 10A, F,* n* = 3, one-way ANOVA, ^***^p < 0.001 vs Control group; ^#^*p* < 0.05, ^##^*p* < 0.01 vs STZ group).

### Zhenzhu Tongluo pills can relieve the increase of apoptosis in DRG of diabetic peripheral neuropathy rats

**Fig. 11:**
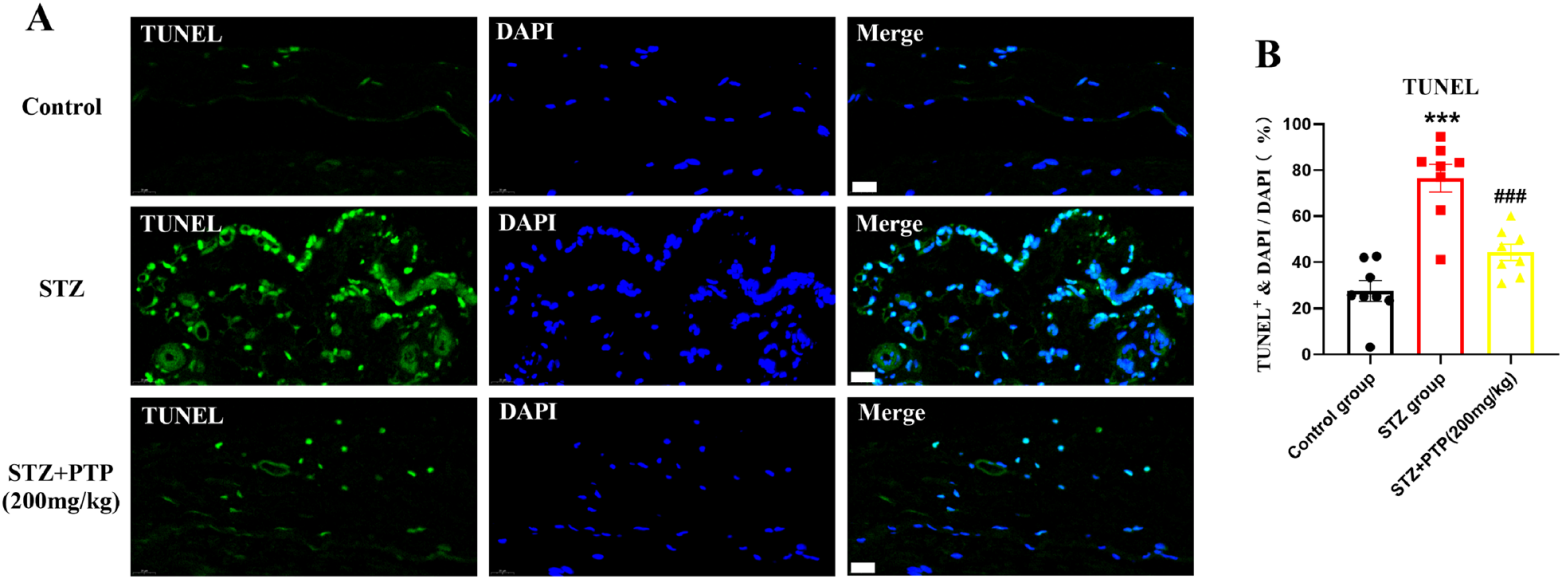
Zhenzhu Tongluo pills can significantly improve the increased apoptosis in DRG of diabetic peripheral neuropathy rats. **A** TUNEL staining diagram (Scale bar = 20 μm); **B** Zhenzhu Tongluo pills could significantly improve the increased apoptosis in DRG of diabetic peripheral neuropathy rats (*n =* 8, ^***^p < 0.001 vs Control group; ^###^*p* < 0.001 vs STZ group)

The above studies showed that the dose of Zhenzhu Tongluo pills (200 mg/kg) was effective and stable, so the molecular index of this dose was selected to verify the effectiveness of Zhenzhu Tongluo pills. TUNEL staining was used to observe the apoptosis of DRG cells in different groups. Compared with Control group, the TUNEL positive staining rate in DRG of rats in STZ group was significantly increased (Fig. 11A, B,* n *= 8, one-way ANOVA, ^***^p < 0.001 vs Control group). Compared with STZ group, Zhenzhu Tongluo pills treatment (STZ + PTP; 200 mg/kg) significantly decreased the positive rate of TUNEL staining in DRG (Fig. 11A, B,* n* = 8, one-way ANOVA, ^###^*p* < 0.001 vs STZ group). The above results indicate that Zhenzhu Tongluo pills can play an effective anti-apoptotic role.

### Zhenzhu Tongluo pills can relieve sciatic nerve injury in diabetic peripheral neuropathy rats

One of the reasons for hyperalgesia in diabetic peripheral neuropathy rats is the obvious injury of sciatic nerve. The above research results indicate that relieving inflammation and modulating the expression of various signaling pathway protein molecules are the possible mechanisms of relieving pain by Zhenzhu Tongluo pills in diabetic peripheral neuropathy rats. However, does Zhenzhu Tongluo pills have a protective effect on sciatic nerve injury in STZ rats? We do not know. Therefore, in this part of the study, HE staining and electron microscopy were used to observe the morphological characteristics of sciatic nerves in different groups.

#### HE staining showed that Zhenzhu Tongluo pills could alleviate sciatic nerve structural injury in diabetic peripheral neuropathy rats

**Fig. 12:**
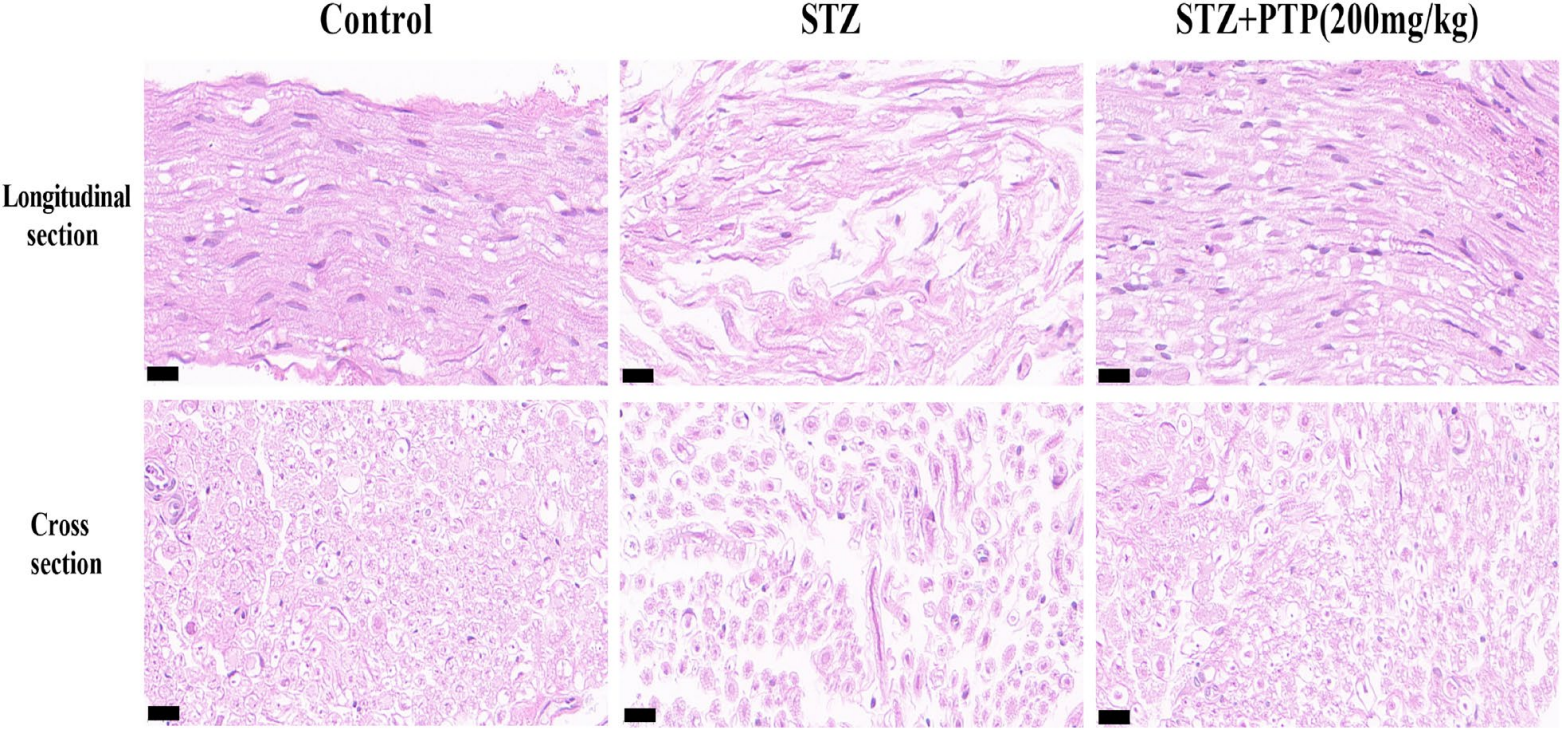
HE staining showed that Zhenzhu Tongluo pills could significantly improve sciatic nerve injury in diabetic peripheral neuropathy rats (Scale bar = 20 μm)

HE staining was performed on the longitudinal and transverse sections of sciatic nerves of rats in different groups to observe the tissue morphology. In Control group, the nerve fibers of rats were normal in size, dense and uniform in distribution, uniform in myelin staining, and axons in the myelin were clear without swelling or atrophy (Fig. 12). In STZ group, the nerve fibers were loosely arranged, myelin edema was uneven, and even demyelination occurred, and axons were significantly changed (Fig. 12). After treatment with Zhenzhu Tongluo pills, the pathological damage of nerve fibers was significantly improved, and the arrangement of nerve fibers was more orderly and the staining density was uniform (Fig. 12).

#### Electron microscope showed that Zhenzhu Tongluo pills could alleviate sciatic nerve structural injury in diabetic peripheral neuropathy rats

**Fig. 13:**
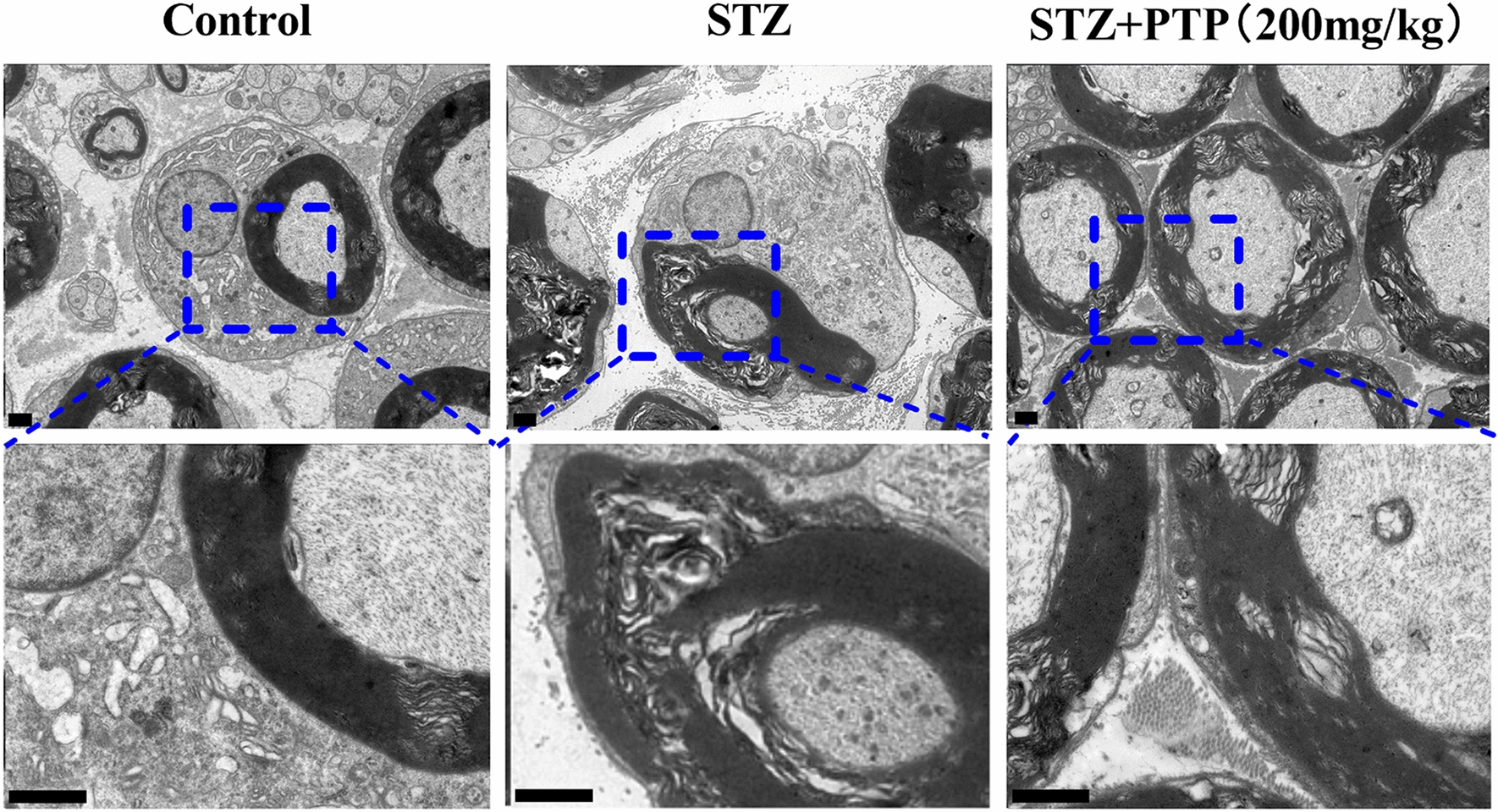
Electron microscope showed that Zhenzhu Tongluo pills could significantly improve sciatic nerve injury in diabetic peripheral neuropathy rats (Scale bar = 1 μm)

Transmission electron microscopy (TEM) was used to observe the ultrastructure of the transverse section of sciatic nerve in different groups. In Control group, the myelin sheath structure of myelinated nerve fibers was complete, dense and neatly arranged, and the myelin plate presented a concentric circular appearance with intact axons (Fig. 13). In STZ group, myelin lamellar separation, axon atrophy and even demyelination of myelinated nerve fibers occurred (Fig. 13). Zhenzhu Tongluo pills treatment (STZ + PTP; 200 mg/kg) significantly reduced demyelination and axon damage, and enhanced myelin regeneration, showed almost well-organized myelin nerve fibers (Fig. 13).

## Discussion

### Hyperglycemia, decreased weight gain, hyperalgesia, damaged nerve structure and abnormal sensory nerve conduction function were significant features of STZ-induced diabetic peripheral neuropathy rats

At present, the establishment of diabetic rat models in preclinical studies is mainly achieved by intraperitoneal injection of STZ. A single intraperitoneal injection of STZ can be used to construct an animal model of type 1 diabetes mellitus. In the animal model of type 2 diabetes, the combination of high-sugar and high-fat feeding and low-dose STZ causes insulin resistance and islet damage in experimental animals, which is the biggest difference from the animal model of type 1 diabetes. For both type 1 and type 2 diabetes, common physiological markers, such as elevated blood glucose and body weight loss, are prominent features. In addition, due to the damage of peripheral nerves caused by hyperglycemia, hyperalgesia is often associated with diabetes patients or laboratory animals. Li et al. induced a rat model of type 2 diabetes by intraperitoneal injection of low-dose STZ combined with a high-fat diet. They found that the rat model of type 2 diabetes maintained a sustained high level of blood glucose and decreased body weight gain for at least six weeks after STZ injection. In addition, through the detection of mechanical pain, thermal pain and sciatic nerve conduction velocity, they further confirmed the successful construction of diabetic peripheral neuropathy rat model [7]. A number of other studies established a rat model of type 1 diabetes by a single intraperitoneal injection of high dose of STZ, and they found that the blood glucose level was significantly increased after at least 72 h of STZ injection and could be maintained for a long time [8–11]. Body weight gain was also significantly reduced compared with the control group [9–11]. Rats in the model group showed significant pain sensitization to mechanical, thermal and cold stimuli [8, 9, 11]. In this study, we constructed a rat model of diabetic peripheral neuropathy by a single intraperitoneal injection of STZ (60 mg/kg). We found that the blood glucose level of rats in the model group increased significantly after 3 days of STZ injection, while the body weight gain was decreased significantly after 7 days of STZ injection. In addition, hyperalgesia, damaged nerve structure and abnormal sensory nerve conduction function were also observed after STZ injection. These results suggest that the rat model of diabetic peripheral neuropathy has been successfully constructed. Through chronic treatment of Zhenzhu Tongluo pills, we found that it did not affect the blood glucose level of diabetic rats, but it could significantly improve the body weight loss of STZ rats. This is not surprising, as a previous study on the pharmacological and toxicological evaluation of Zhenzhu Tongluo pills has found that Zhenzhu Tongluo pills can increase weight in normal rats [12]. In addition, we found that Zhenzhu Tongluo pills significantly improved the hyperalgesia, damaged nerve structure and decreased sensory nerve conduction velocity in STZ rats, which also confirmed the neuroprotective function of Zhenzhu Tongluo pills found in previous clinical studies.

### Zhenzhu Tongluo pills may play a neuroprotective function through its anti-inflammatory and anti-apoptotic effects

Zhenzhu Tongluo pills is one of the representative traditional Mongolian medicine, which has been used in clinical treatment of hemiplegia and other diseases. In recent years, the results of clinical studies show that Zhenzhu Tongluo pills play a good role in the treatment of a variety of nervous system diseases and other diseases. One study reported that Zhenzhu Tongluo pills significantly improved the degree of neurological impairment in patients with cerebral infarction and its clinical efficacy [1]. Another study reported efficacy and short course of Zhenzhu Tongluo pills in treating the sequelae of ischemic stroke [2]. In addition to the single use, many studies combined Zhenzhu Tongluo pills with other treatment methods in clinic, which has played a good therapeutic effect. Hao et al. combined Huatan Tongluo Decoction with Zhenzhu Tongluo pills to treat cerebral infarction, and they found that this methos can significantly improve the therapeutic effect and neurological function [3]. Wang et al. combined Zhenzhu Tongluo pills with external application of Qingpeng ointment to treat bruising bone disease, and found that it could improve the effective rate of treatment, reduce the incidence of adverse reactions, and alleviate symptoms such as swelling, pain, ecchymosis and dysfunction, with high safety [4]. Combined Zhenzhu Tongluo pills with acupuncture, Wang et al. reported a remarkable effect in the treatment of cerebral infarction, which can effectively relieve inflammation and improve nerve function [5]. Du et al. also found that combined Zhenzhu Tongluo pills with rotator cuff function exercise can improve the clinical efficacy of chronic rotator cuff injury, improve shoulder joint function, enhance shoulder joint motion, and relieve patients’ pain [6]. The results of these different clinical studies suggest that Zhenzhu Tongluo pills alone or combination with other therapeutic means plays an important role in neuroprotection in clinic. However, most clinical studies are observational studies, which only observe the effect without explaining the mechanism.

Although network pharmacological analysis of Zhenzhu Tongluo pills has found that PI3K–AKT, MAPK and NFκB signals may be important pathways for the function of Zhenzhu Tongluo pills, further study is missing. In this study, we constructed a rat model of diabetic peripheral neuropathy, combined with behavioral, morphological, functional and other molecular biological techniques. The possible mechanism of the neuroprotective function of Zhenzhu Tongluo pills was investigated. Our results suggest that the protective effect of Zhenzhu Tongluo pills on hyperalgesia in diabetic peripheral neuropathy rats is closely related to its anti-inflammatory and anti-apoptotic functions. In fact, many previous studies have found that the analgesic effects of proprietary Chinese medicines or components from plant extracts in rat models of diabetic peripheral neuropathy may be related to anti-inflammatory effects. For example, kaverol extracted from mustard seed reduced the expression of TNF-α, TGF-β and IL-1β in sciatic nerve [11], jinmai Tong regulated the inflammasome in DRG [13], koumine and loganin regulate inflammation at the spinal cord level [10, 14] and treadmill training combined with insulin achieved anti-inflammatory effect [9, 15]. In summary, combined with the results of our study, the anti-inflammatory effect of Zhenzhu Tongluo pills is one of the mechanisms to exert its analgesic effect.

Our results in this study confirmed that Zhenzhu Tongluo pills can indeed regulate the expression of PI3K–AKT, MAPK, NFκB and other related molecular proteins, suggesting that the neuroprotective function of Zhenzhu Tongluo pills may be realized through the above signaling pathways. In fact, previous studies have shown that different drugs alleviate diabetic peripheral neuropathy through regulating these signaling pathways. For example, empagliflozin could regulate the expression of MAPK and NFκB pathway and finally reducing inflammatory response [16], Baimai cream has good efficacy in regulating the expression of PI3K/AKT and MAPK signaling pathways [7], and diosgenin could regulate the expression of NFκB pathway and thus alleviating inflammatory response [17]. Combined with our study, we have reason to speculate that multi-target action may be the dominant feature of Chinese medicine therapy.

### Zhenzhu Tongluo pills can improve the structural and functional damage of sciatic nerve in diabetic peripheral neuropathy rats

Pathological lesion of sciatic nerve is a significant feature of diabetic peripheral neuropathy rats. Under a light microscope, the sciatic nerve often shows vacuol-like degeneration of medullated nerve fibers and axonal swelling. Under electron microscope, it showed serious demyelination. In this study, on one hand, the longitudinal and transverse sections of sciatic nerve were observed by HE staining, and it was found that the sciatic nerve of STZ group rats was characterized by loose nerve fiber arrangement and uneven myelin edema. On the other hand, the results of transmission electron microscopy also showed the separation of myelin lamella, axon atrophy and demyelination of myelinated nerve fibers in the STZ group. In addition, the results of sciatic nerve conduction velocity also showed that the sciatic nerve conduction velocity was significantly damaged in the STZ group. These results suggest the successful construction of STZ-induced diabetic peripheral neuropathy rat model both structurally and functionally.

Many previous studies have confirmed that different drugs can also improve the sciatic nerve structure injury when improving the peripheral injury of diabetic peripheral neuropathy rats. Abdelkader et al. found the improvement effect of empagliflozin on sciatic nerve structural damage in diabetic peripheral neuropathy rats by HE staining, toluidine blue staining and projective electron microscopy [16]. Li et al. also found that Baimai cream had a protective effect on the structural and functional damage of sciatic nerve in rats with diabetic peripheral neuropathy by electron microscope and evaluation of sciatic nerve conduction velocity [7]. Other studies have also found that extracts from *Labisia* *pumila* or *Datura* *innoxa* can reduce the structural damage of sciatic nerve [8, 18]. In this study, on one hand, we found that Zhenzhu Tongluo pills had a significant improvement on the structural damage of sciatic nerve in rats with STZ-induced diabetic peripheral neuropathy, which was specifically manifested in the structural effects of nerve fiber arrangement, axon injury and demyelination. On the other hand, Zhenzhu Tongluo pills can also relieve STZ-induced reduction of sciatic nerve conduction velocity functionally. These findings further confirm that Zhenzhu Tongluo pills has significant neuroprotective function from the structure and function of nerves.

In this study, although we preliminarily explored some mechanisms of the neuroprotective effect of Zhenzhu Tongluo pills in an STZ-induced diabetic peripheral neuropathy rat model, it is still insufficient. In fact, in the future, it is necessary to build relevant animal models of nervous system diseases, such as animal models of cerebral ischemia reperfusion injury and cerebral hemorrhage, etc., combined with behavioral tests of motor function, cognitive function, memory function, etc., to explore the central mechanism of neuroprotective effect of Zhenzhu Tongluo pills at the brain level.

## Data Availability

The data sets used and/or analysed during the current study available from the corresponding author on reasonable request.
